# Can Schools Support HIV/AIDS-Affected Children? Exploring the ‘Ethic of Care’ amongst Rural Zimbabwean Teachers

**DOI:** 10.1371/journal.pone.0146322

**Published:** 2016-01-20

**Authors:** Catherine Campbell, Louise Andersen, Alice Mutsikiwa, Claudius Madanhire, Constance Nyamukapa, Simon Gregson

**Affiliations:** 1 Department of Social Psychology, The London School of Economics and Political Science, London, United Kingdom; 2 Biomedical Research and Training Institute, Harare, Zimbabwe; 3 School of Applied Human Sciences, University of KwaZulu Natal, Durban, South Africa; 4 Department of Infectious Disease Epidemiology, Imperial College School of Public Health, London, United Kingdom; Leibniz Institute for Prevention Research and Epidemiology (BIPS), GERMANY

## Abstract

How realistic is the international policy emphasis on schools ‘substituting for families’ of HIV/AIDS-affected children? We explore the ethic of care in Zimbabwean schools to highlight the poor fit between the western caring schools literature and daily realities of schools in different material and cultural contexts. Interviews and focus groups were conducted with 44 teachers and 55 community members, analysed in light of a companion study of HIV/AIDS-affected pupils’ own accounts of their care-related experiences. We conceptualise schools as spaces of engagement between groups with diverse needs and interests (teachers, pupils and surrounding community members), with attention to the pathways through which extreme adversity impacts on those institutional contexts and social identifications central to giving and receiving care. Whilst teachers were aware of how they might support children, they seldom put these ideas into action. Multiple factors undermined caring teacher-pupil relationships in wider contexts of poverty and political uncertainty: loss of morale from low salaries and falling professional status; the inability of teachers to solve HIV/AIDS-related problems in their own lives; the role of stigma in deterring HIV/AIDS-affected children from disclosing their situations to teachers; authoritarian teacher-learner relations and harsh punishments fuelling pupil fear of teachers; and lack of trust in the wider community. These factors undermined: teacher confidence in their skills and capacity to support affected pupils and motivation to help children with complex problems; solidarity and common purpose amongst teachers, and between teachers and affected children; and effective bridging alliances between schools and their surrounding communities–all hallmarks of HIV-competent communities. We caution against ambitious policy expansions of teachers' roles without recognition of the personal and social costs of emotional labour, and the need for significant increases in resources and institutional recognition to enable teachers to adopt support roles. We highlight the need for research into how best to create opportunities for teacher recognition in deprived and disorganised institutional settings, and the development of more culturally appropriate notions of caring.

## Introduction

How can schools facilitate the inclusion and well-being of HIV/AIDS-affected children in sub-Saharan Africa? In the region 21.8 million adults (aged 15 and over) and 2.9 million children (14 and under) are living with HIV, and 15.1 million children have lost one or both parents to AIDS [1,2]. Many youths are growing up with sick or absent parents, with extended family often at breaking point [3]. International policies increasingly advocate an enhanced role for schools in caring for HIV/AIDS-affected children [4,5]. Expanding the traditional role of African schools, from preparing young people for the workplace to “substituting for families”, requires the development of an explicit ethic of care to guide and support the education sector however [6]. To what extent is this policy expectation realistic? Are teachers prepared, willing and able to take on the pastoral care and social protection of children with complex problems? To what extent does the basis for such an ethic of care exist?

We explore these issues through a study of understandings and practices of care by teachers in rural Zimbabwe, locating these issues within the complex spaces of engagement between children, teachers and surrounding community members that constitute the school environment. Economic and political instability, dwindling teacher numbers, and the withdrawal of many non-governmental organisations (NGOs) has led to reduced support for Zimbabweans affected by HIV/AIDS, a fall in the status of the teaching profession and a dearth of human and financial resources for schools-based HIV/AIDS responses [7–9]. Given wider international debates about the role of local communities in responding to social problems–in contexts of reduced welfare support from governments and the contraction of international development funding–Zimbabwe provides a particularly productive site for exploring the potential for indigenous local responses to HIV/AIDS in marginalised settings that lack substantial external help or support [10].

UNESCO’s *Good Policy and Practice on HIV and AIDS in Schools* report [5] is one of many international policy documents that maps out a caring role for schools. Their account of this role has five dimensions, the first aligned with the more familiar role of schools in imparting information and skills, and the other four advocating an expanded role for schools in relation to care and support. The five roles are: (i) the provision of HIV/AIDS related education for all children, (ii) ensuring the access and enrolment of HIV/AIDS-affected children, (iii) providing them with psycho-social support and counselling, (iv) assisting them to access the nutritional, health and medical services that they need, and (v) building partnerships between schools, local communities and relevant health and welfare agencies to support up in these roles. Many such documents frame these tasks within the wider goal of ‘empowering’ HIV/AIDS-affected children to express their needs, through e.g. establishing children’s committees within a children’s rights framework [11,12]. Acknowledging that teachers are also vulnerable to HIV/AIDS (in Zimbabwe an estimated one in three teachers are HIV positive [9]), policies also advocate that schools offer similar care and support to HIV/AIDS-affected teachers [4,5].

A large literature exists on the first policy focus, HIV education in schools [13], which falls outside the scope of this particular paper. Schools-based HIV/AIDS interventions have been criticised for prioritising HIV education and prevention at the expense of the care and support needs of under-served children who are already infected or affected [14]. We contribute to filling this gap through focusing on the other dimensions of international school-related HIV/AIDS policy: facilitating school enrolment, providing psycho-social support, assisting with nutrition and access to health services, and building alliances with outside agencies, using child empowerment-based approaches. A growing handful of articles report on NGO-driven interventions to train southern Africa teachers to provide care and support for HIV/AIDS-affected children [15,16]. Little attention is given to the wider context of these interventions, however, in terms of how they might fit into the wider ethos of schools or teacher willingness and preparedness to take on these expanded roles. Thus, rather than focusing on specific activities or interventions in schools, this paper responds to Ansell’s [6] call for greater attention to the wider ethic of care amongst teachers. This includes attention to teachers’ own understandings of HIV/AIDS-affected children’s needs, their commitment and readiness to take on caring roles, and how this readiness is impacted by wider contexts of thin or patchy NGO and public sector support for schools.

International health and education policies are often formulated by experts in northern settings, resonating poorly with the realities of target communities [17]. Writing about the psycho-social challenges facing HIV/AIDS-affected children in Zimbabwe, Parsons [18] highlights the gaps between how children and their carers understand and experience their life situations, and what he calls the ‘Western social and emotional lexicon’ that is used to describe them. Studies also increasingly point to the gap between international policies on the role of schools in the HIV response and the daily realities of teachers and learners in South Africa [19], Malawi and Kenya [20] and Zimbabwe [14,21]. This gap forms the context of this paper.

Our primary goal in this multi-disciplinary paper is to advance the theory and practice of social policy relating to the education, health and welfare of vulnerable children in extremely difficult settings [22]. The support needs of children affected by HIV/AIDS in Zimbabwean schools serve as a particularly compelling illustration of the challenges of supporting children in adversity around the world, given the high degree of suffering of the children and the extent of material deprivation and symbolic stigma framing Zimbabwean school settings [18]. We also seek to advance academic understandings of the social relationships and identifications that underpin the act of caring in extreme settings, as well as the associated empirical literatures on the ‘ethic of care’ in schools and on so-called ‘caring schools’. Our outline of the conceptual tools we used to frame our research design and data collection, and to interpret our findings, seeks to counter critiques of the existing literature on school support for vulnerable children as piecemeal and descriptive rather than analytical. This outline also addresses calls for explicit conceptual frameworks that start to systematise existing knowledge. Such frameworks should move beyond description to point to new ways of being, seeing and doing that pay greater attention to the ‘goodness of fit’ between policy and real life [23].

### Conceptual framework

How do social spaces such as schools impact peoples’ opportunities for health and well-being? We drew on complementary concepts from the fields of education, human geography and social psychology in developing the conceptual frame for our study. These included the concept of *care as a contextualised social relationship*, a focus on the *social spaces of engagement* in which care is enacted–and the opportunities these spaces offer social participants (such as teachers and children) for the forms of *social recognition and identity* that underpin the possibility of caring relationships. The concept of an ‘ethic of care’ in schools [24] is widely used in US research. ‘Caring teachers’ have been found to play a key role in the enrolment and progress of vulnerable students, with empathy cited as the hallmark of a caring relationship [25]. For Noddings [24], a caring relationship is characterised by an attitude of “receptivity, relatedness and responsiveness” by the “one-caring” towards the “cared-for”, and a relationship of close identification by the former with the latter:

*“When I see the reality of the other as a possibility for myself*, *then I care*. *This involves apprehending the possibility of the other’s reality as my own—once I do this*, *I am impelled to act as though on my own behalf*, *but on behalf of the other*. *I act to help the other through seeking to eliminate the intolerable*, *to reduce the pain*, *to fill the need*, *to actualise the dream*.*”* [24]

Noddings is particularly interested in the wider contexts that enable or limit the possibility of caring. According to Whitney [26] a caring teacher is one who works to construct caring relationships with learners that are not linked to classroom performance; shows empathy for ‘at risk’ students; creates opportunities for students to develop a positive sense of self; creates a positive classroom experience including attentiveness and responsiveness to student needs; negotiates student-teacher conflict in a calm way that allows the student to ‘save face’; and practices flexibility in allowing students to work at different speeds and on different material where necessary.

In Europe, similar values are echoed by the social pedagogy movement [27], which emphasises not only teacher-learner relationships within the school, but also the need for schools and their surrounding communities to collaborate in providing children with the care they need in order to become empowered, happy and healthy citizens. This resonates with Freire’s [28] ideals of education based on mutual respect and dialogue between teachers and learners, with learner views taken seriously by responsive and respectful teachers, who work hard to see things form the learners’ point of view, and to tailor their input to learners particular needs–framed by an interest in ‘empowering’ learners to become confident agents in their lives, rather than victims of social circumstances.

Whilst the notion of the caring school is increasingly being taken up in the grey literature in southern Africa [29] more systematic attention needs to be paid teachers’ own attitudes to these roles, and the social contexts that promote or hinder the likelihood that teachers would be able or willing to care for students in this way. Furthermore the research literature often tends to focus on particular groups within the school, often teachers *or* pupils, or on particular aspects of teacher-pupil relationships, with fewer case studies locating caring within a moore holistic view of the wider range of engagements constituting the school environment.

To occupy this gap, we conceptualise schools using Massey’s [30] notion of the ‘social spaces’ constructed in and through relationships between diverse groups, in our case children, teachers, head-teachers, parents or guardians, and community members. Such groups often have different priorities and interests and diverse understandings of common daily challenges and optimal responses. Divergent understandings of problems within and between groups may be a source of strength or weakness. Massey takes an optimistic view of the existence of differing worldviews within a particular social space, seeing these as a source of growth and transformation [30]. Our case study below presents a less optimistic picture of often irreconcilably conflicting needs and interests within our social space of interest. We will argue that differences amongst the priorities of teachers, parents/carers and pupils often hinder rather than facilitate an effective ethic of care in many (though not all) day-to-day school situations.

Social spaces are not neutral containers for behaviour, but contexts that afford or enable particular forms of representation, communication and action, and the social identities that emerge from them [31,32]. In this regard, identity and space are co-emergent processes. Identities (e.g. of both children and teachers) are shaped by three forms of *recognition* that lie at the heart of well-being and of caring relationships: inter-individual empathy and respect, the recognition of one’s value to one’s community, and the formal legal recognition of one’s right to respect and health [33]. What opportunities does the school-in-context present for the recognition of teachers and pupils in our study? How does the presence or absence of such recognition enable or limit the possibility of caring relationships? What opportunities exist for reconfiguring these in more positive and enabling ways? These questions guide our study below.

### Context of study

We draw on findings from a larger multi-method study of school support for the inclusion and well-being of children affected by HIV/AIDS in rural Zimbabwe. The project received ethical approval from the Medical Research Council of Zimbabwe (MRCZ/A/1661) and the Research Ethics Committee at the London School of Economics. Informed written consent was gathered from all research participants on condition that the name of the study districts and identities of schools and participants would not be revealed.

Seventy percent of Zimbabweans live in rural areas [34], home to the majority of orphans and vulnerable children [35]. HIV prevalence is 15.8% amongst adults (aged 15–49) [35], and 2.7% amongst children (under 14) [35], and 19% of children have lost one or more parents, mostly due to AIDS-related illness [36]. ART coverage for children and adults stands at 76% for adults and 46% for children [37], though many barriers challenge optimal adherence [38].

Poverty is rife and many local people hope for food aid from government and international organizations although supplies fluctuate. Children often play a key role in sustaining households with sick or absent adults, through caring for young, sick or elderly, conducting chores like cleaning, cooking, collecting firewood and water, and contributing to income generation through farming. HIV remains stigmatised, accompanied by bullying and social rejection of HIV/AIDS-affected children [39–41]. They may often be vulnerable to sexual and other forms of abuse by adults including teachers. In such context, the suffering of children is often extreme, with Parsons [18] saying that only the literature on the holocaust comes close to matching the degree of exclusion, fear and exhaustion suffered by many Zimbabwean HIV/AIDS affected children. The 2000s saw economic and political instability in Zimbabwe. Inflation rose from 1281% in 2006, to 231,150,888% in 2008, curtailing the government’s ability to pay teachers regular salaries. Schools effectively ceased to function, and teachers lost morale and confidence in their profession. The Zimbabwe dollar was replaced with a multi-currency system early in 2009 with the situation stabilising somewhat since then, but teacher salaries remain low. At the time of our study in 2012 salaries averaged approximately $400 per month, which placed them below the “breadline” of $500 required just to feed a family of five [42,43]. Many teachers ‘moonlighted’ to earn money through other means, with an increase in teacher absenteeism and drinking [9]. The status of teaching continues to decline, with a brain-drain to neighbouring countries offering better wages and conditions of service, and fewer young people joining the profession [43,44].

Companion papers from our larger multi-method study documented schoolchildren’s own accounts of challenges facing HIV/AIDS-affected peers and of school support [39–41]. Children spoke of poverty, hunger, unhealthy living conditions and stigma. They reported neglect and abuse by adults, heavy household duties and caring responsibilities limiting school attendance and progress, and lack of school essentials (uniforms, books, pens). They emphasised that these challenges caused immense emotional distress including physical exhaustion, fear and sadness, bullying and social isolation. Schools were seen to provide limited help through occasional material assistance (books, pens) from teachers and school fees from NGOs. School attendance was said to provide children with distraction from their home problems, and opportunities to construct a positive sense of self and hope for the future. Against the backdrop of this companion study of children’s own accounts of their experiences, the current paper explores the schools-based ‘ethic of care’ from the perspective of teachers and adult community members. Conceptualising schools as the spaces of engagement among teachers, communities and children themselves, we draw on these companion studies [39–41] of children’s own voices to contextualise our findings.

## Methodology

In-depth interviews were conducted with 22 teachers (6 male school principals, as well as 8 male and 8 female classroom teachers) and 3 mixed-gender focus groups were conducted with a further 22 teachers, all employed by 6 schools (half primary and half secondary) in our province of interest. The average age of the classroom teachers interviewed was 40 years and the average age of the principals was 50 years. A further 7 mixed-gender focus group discussions were conducted with 55 members of the communities surrounding each school (aged 30 to 60), many of them parents, and two further interviews were conducted with local NGO representatives. Interviews averaged 65 minutes and focus groups 76 minutes, all in participants’ home language.

The interviews and focus groups were conducted by 3 Zimbabwean fieldworkers, two females and one male. Both female fieldworkers had undergraduate social science degrees. The male fieldworker (CM, co-author of this paper) managed the fieldwork team and had a master’s level qualification in social work and over 10 years’ experience as a qualitative researcher. He worked closely with the second author of this paper (LA), a Danish anthropologist with three years of research and NGO management experience with HIV/AIDS-affected children in Africa. Over a three-week period, LA and CM conducted a detailed fieldwork training program with the two female fieldworkers, which included qualitative research methods training, extensive discussion of the topic guides, and role playing to practice interviews and focus groups.

The fieldworkers had worked on previous research involving the schools, and had good links with each headmaster. Each head was asked to identify three teachers in addition to himself, including at least one man and one woman, for interview. It is likely that heads selected teachers most likely to make a favourable impression, with a definite bias towards more senior teachers in our sample. Notwithstanding, we believe participants gave a frank account of the limitations of their schools’ HIV responses. Participants were approached in person by the researchers, who explained the study’s purpose to them and invited them to take part. All those approached agreed to participate. The interviews were conducted in the schools or homes of participants, for maximum participant privacy and comfort.

Interviews and focus groups were audio-taped with signed consent, and translated and transcribed by fieldworkers. Teacher topic guides focused on (i) the impact of HIV/AIDS on pupils, (ii) manifestation of HIV/AIDS in school settings, (iii) teacher responses, and (iv) factors influencing teacher responses. Community topic guides focused on (i) community perceptions of the impact of HIV/AIDS on local children; (ii) community efforts to support affected children; (iii) factors impacting the quality of this support; and (iv) community perceptions of the role of the school.

**Fig 1:**
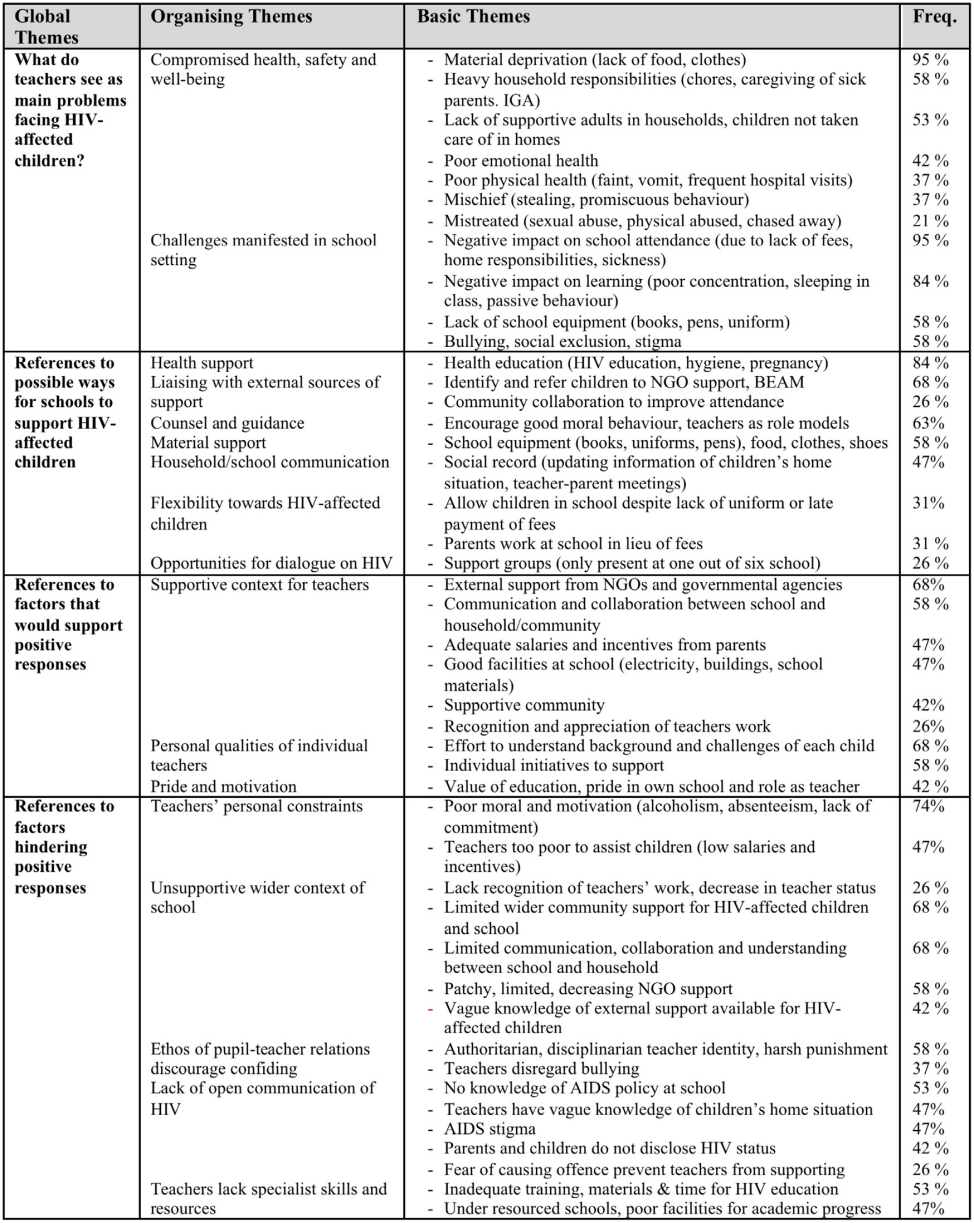
Analysis of teacher data.

**Fig 2:**
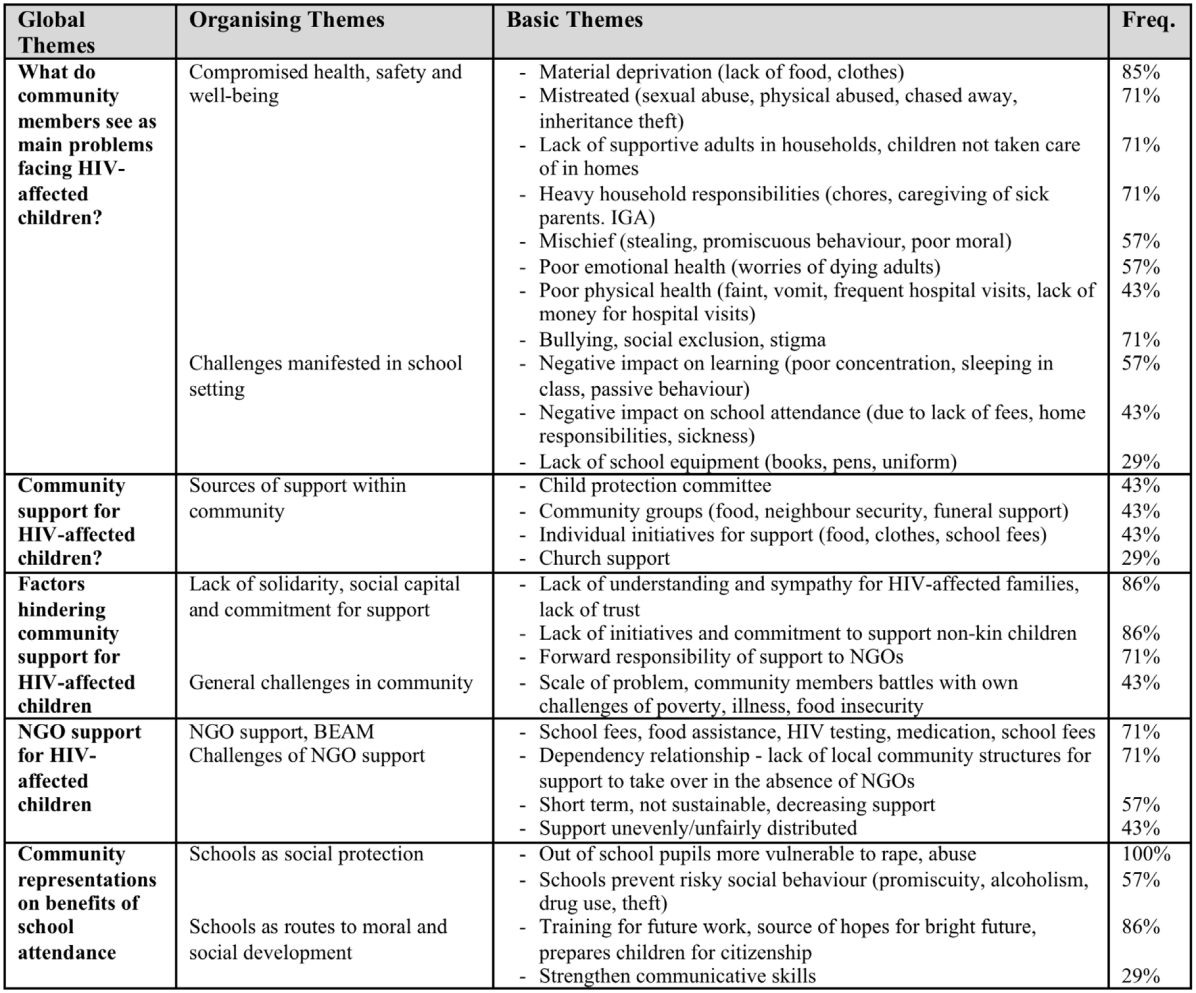
Analysis of community data.

The first and second authors analysed the data through ‘summarising content analysis’ [45]. This analysis involved close reading and re-reading of transcripts, alongside intensive dialogue over several weeks about themes arising from the data in relation to the key topics of interest: teacher and community understandings of the needs of AIDS-affected children, how teachers were responding to AIDS-affected children, and factors that facilitated or hindered teachers in providing care for these children. By the end of this period they developed a coding system to best summarize the content of the data and were able to code the data, using the qualitative software NVIVO. Coding frames (Figs 1 and 2) document the content and frequency of themes. We draw selectively on this information in discussing the ethic of care in our study schools. In the findings section, below, informants are identified as follows: T = teacher, H = headmaster, C = community member. Schools A, B and C are primary schools. Schools D, E and F are secondary schools.

## Findings

### Teachers’ representations of care

#### Problems facing HIV/AIDS-affected learners

Teacher’s accounts of problems facing children closely echoed children’s own accounts in the companion study: lack of basic needs (especially food and clothes), poor physical health, heavy home responsibilities (chores and caring), stigma and bullying and poor emotional health:

*The child does not have a balanced diet*. *He may come to school without having eaten because the person who should provide for him is bedridden*, *TD02*

*Affected children don’t wear shoes*. *Some sleep in class because they are hungry*. *TA03*

*Yesterday I asked children to write an essay on advising a friend whose parents had HIV*, *and a child started to cry*. *I didn’t know how to deal with this*. *The child didn’t want to disclose anything so I was unable to help*. *Often it’s clear a child is not happy*. *But if they disclose their situation*, *other children won’t play with them or share food for fear of contracting HIV*. *TE02*.

HIV/AIDS-affected children often experienced disrupted school attendance and poor performance.

*There is a noticeable difference–parents of children unaffected by HIV do the chores for them but HIV-affected children have greater responsibilities*. *These divert them from focusing on their education… Even when the child is at school she won’t have peace of mind like others with healthy and living parents*. *TD02*

#### Teacher responses

Teachers’ accounts suggested there were no systematic or united responses to supporting children. They often suggested actions teachers might take in principle, emphasising that teachers should make every effort to understand a child’s circumstances, and to do their best to assist them in tackling their problems. They advocated that teachers should act like ‘loving parents’ and that ‘schools should serve as children’s ‘second homes’.

*The first thing you should show your pupils is love*. *Teachers should be friendly to the children*. *They should be polite*. *They should be patient*. *They should be tolerant to the children just like a good parent*. *(TD02)*

*It is the duty of the class teacher to know the background of every child*, *even if you have forty-five pupils*. *They are yours*. *You are the parent*. *(TD03)*

Despite recognising the potential for schools to play a caring and protective role, interviews suggested teachers had seldom or never actioned this.

*We haven’t done much to give support*. *TD02*

*You should create a bond with an HIV-affected child … Go an extra mile to help them*… *Once you know their backgrounds and they trust you*, *then you can start teaching… However in most cases the problems of HIV-affected children are ignored*. *A teacher may be teaching forty pupils*, *they tend to concentrate on children participating most actively in lessons*. *For those not participating*, *teachers don’t go that extra mile to find out what’s really happening to that child*. *TF02*

The responses teachers did speak of were low profile, usually isolated and one-to-one acts of individual kindness. These included an isolated reference to a teacher making a home visit to see why a child had been absent for so long; occasional referrals of children to outside NGOs, mainly to help with fees; providing children with books, pens or uniforms; advice on diet; assistance with medication; flexibility in allowing the child to stay in school despite lack of uniform; exempting very sick children from heavy school chores, and allowing impoverished parents or guardians to clean the school in lieu of payment of fees. Such support was overwhelmingly material or practical, with virtually no references to emotional support. Even occasional teacher references to ‘counselling’ tended to refer to instruction and discipline rather than empathetic listening or comforting.

*We sometimes do guidance and counselling*, *encouraging them to abstain from sex; telling them about the negative implications of sexual activities TD03*

Some teachers referred to HIV/AIDS-related bullying. References to intervening involved punishing the bully, however, with no discussion of providing emotional support to the victim.

#### Factors supporting positive teacher responses

What latent resources might be mobilised by those seeking to enhance school responses? Despite low payments and declining status, several teachers expressed professional pride in the value of schooling, some were proud of their schools, and others derived positive self-esteem from the role of teacher.

*I grew up wanting to become a teacher*. *I never wanted any other profession*, *TD02*

*I am happy because this school is one of the best in the district*. *TA01*

Kind responses by teachers were explained in terms of personal qualities of individuals, with a general representation of older teachers as kinder than younger ones, and primary school teachers kinder than secondary. Other positive factors were support from external NGOs and the community when these were available. Teachers occasionally made reference to the existence of school systems, which though seldom implemented, provided formal bureaucratic means to track child attendance, one being ‘child social records’ aiming to record a child’s personal circumstances and attendance, enabling teachers to follow up long-absent children.

#### Factors hindering positive teacher responses

The bulk of interviews and focus groups with teachers referred to constraints on their ability and motivation to support HIV/AIDS-affected children. Frequent reference was made to the unsupportive nature of the wider community context.

*There isn’t a place where we can say they (HIV-affected children) can get direct support*. *There are no structures in the school or community we could point to*, *or organisations or parents meetings to focus on the needs of the affected children*. *TC02*

Until recently there had been a highly effective community-based organisation near two of our schools, which had, for a while, received donor funding to assist children with fees. However, it seemed this funding had dwindled. The Basic Education Assistance Model (BEAM), a national government agency for distributing donor funding for fees, especially from UNICEF, had been a vital lifeline for many children, but had recently experienced dramatic funding cuts.

Parental support for the school appeared to be patchy. Teachers reported that some relatively well-off parents resisted paying fees, viewing it as unfair that they should pay regularly when more impoverished parents did not.

*The parents do not want to pay fees before other parents have paid*. *So they will only pay when the children are sent home [threatened with exclusion]*. *When we have AGMs (Annual General Meetings) with parents this issue is raised repeatedly*. *Parents say they are not going to pay school fees this year*, *until other parents are forced to pay outstanding fees for the year before*. *TD02*

This reluctance to pay fees meant that funding was often not available for core school activities. Furthermore, teachers often felt discouraged and undervalued by this lack of parental support.

High AIDS stigma and denial meant that most parents and carers of HIV-positive children were fiercely secretive about their status. Teachers said this stigma-fuelled secrecy made it impossible to acknowledge or respond to a child’s special needs for fear of offending the child and family. This led to situations where, for example, the teacher was not able to refer a seriously ill child for HIV testing and treatment, for fear of offending the child and family by suggesting the child might be HIV-positive, even though the nature and degree of the child’s illness might be obvious.

*I am not sure of anything that the school has done to help HIV affected children*. *Many children have not disclosed their status*, *making it difficult for school heads to offer support because it seems too sensitive*. *We feel that this information must just be brought to our attention by the HIV-affected child… giving them special treatment that they have not asked for may make them feel inferior*. *TE02*

*There is a lack of communication from that child or from the guardians he/ she is living with… You know our culture*, *the things are hidden… Some children do not talk at all*. *They do not disclose what their situations are like*. *They hide such things*. *TC02*

*You might notice the signs and symptoms but it’s hard to ask the child whether he is positive*. *And if we were to ask a child what is bothering him he is not free to open up and tell us that he is HIV positive*. *CA04*

The lack of proper toilets, deteriorating buildings, not enough chairs and tables, and no electricity, telephone or computers in some schools, discouraged many teachers’ confidence in what they had to offer. More directly HIV/AIDS related, teachers’ responses to affected children were also hindered by their view that they lacked specialist skills to support highly distressed or very deprived children, as well as appropriate training, materials and allocated time for HIV/AIDS related education and support work in the schools.

*The Ministry says that AIDS education should be compulsory but in reality it is difficult to slot it on the timetable*. *TD02*

*I just feel that the time allocated for the things to do with HIV/AIDS is too little*. *TA03*

Authoritarian and disciplinarian views of teacher-child relationships also stood in the way of emotional responsiveness by teachers. They tended to view their relationship with children through the lens of discipline rather than caring, associated with harsh punishments, severe beatings and psychological distance. They commented that in a climate of harsh teacher attitudes and excessive corporal punishment, children often feared teachers, making it unlikely that they would confide in them.

*Children often see teachers as lions or tigers*. *The children dread to go to school in the morning fearing the teacher*. *TC02*.

*How will children report sensitive problems to teachers in such state of fear*? *HA01*

Other teachers commented that the severity of their own personal problems often made them ill tempered and intolerant of children that approached them for any reason at all.

Finally teachers felt very undermined by the low levels of recognition they received from the government which they associated with their low salaries, not always paid on time.

*The salaries that we are being given by the government*, *it cannot sustain us*. *So it hurts us so much that we are not given incentives by parents like other communities are able to give teachers*. *TF01*

*I think education is becoming challenging*. *Teachers have to be considered and raised … currently they are not being recognised*. *TF03*

They felt that common knowledge of their predicament also lowered their status in the community. At the time of the research, much local amusement was being generated by a joke about a schoolteacher who had been forced to steal her pupil’s pocket money, because child’s pocket money was now higher than teacher salaries.

#### Case study

A brief focus on a single primary school teacher provides one instance of how some of these broader themes cluster together in the experiences of a single teacher. Mr M (in his 40s) was the most committed teacher in our sample, speaking in the most detail in his interview. He became a teacher in this remote rural area after losing a well-paid business job in Harare, involving loss of income and his previously valued identity as ‘a prosperous person’. However he had made the most of a negative situation, taking pride in the value of education and in the good reputation of his school. He described himself as relatively motivated to help his pupils, saying that he differed from younger teachers, who had less interest in their pupils. He said younger teachers were often strongly opposed to offering non-educational support to pupils, insisting that they were not ‘donors’.

Mr M appeared to be a kind person, who spoke sympathetically of the poverty and desperation of many of his HIV/AIDS-affected pupils, but he had not offered support to such pupils on more than an isolated number of one-off occasions. These included him occasionally taking children who persistently fainted or fell asleep in class to his home for a meal. He talked of visiting the home of a persistently absent pupil and the distress of the sobbing grandmother unable to feed the orphaned grandchildren in her care, prompting him to collect money from other teachers to buy her a bag of maize. He said such action on his part was unusual however, and that he was unable to help in this way very often due to his own poverty. We certainly would not seek to undermine the extremely positive intentions and impacts of such actions in individual cases. However, we would argue that one-off, piecemeal and institutionally isolated efforts do not constitute the type of systematic or comprehensive response that international policy might hope for.

He had a vague memory of HIV workshops to equip teachers to support HIV/AIDS-affected children, but was unable to remember whether he attended these or what he might have learned from them. He spoke of teaching ‘HIV/AIDS awareness’ to his pupils, but when asked about the content, he emphasised that he was ‘not allowed’ to talk about condoms or sex, because discussions of sex with children would encourage ‘experimentation’. He said there was little or no discussion of HIV or AIDS in his school. He was not aware of any parents or carers disclosing that they or a child were HIV-positive for fear of stigma. In a context where one in three teachers are estimated to be HIV-positive, Mr M said he had never known of an HIV-positive teacher disclosing their status, saying this would provoke an extremely negative reaction. He said he was chair of his school’s child protection committee, but insisted there was no bullying in his school (although bullying was repeatedly emphasised by children in his school in our companion study). He said he had never discussed a personal problem with a child because primary school pupils were ‘too young’ to approach a teacher, and because the law prescribed that teachers could only talk to learners in English, which most primary school pupils are not yet able to speak.

In summary, interviews with Mr M and other teachers suggested that, beyond isolated individual acts of kindness, there was no generalised or institutionalised ethic of care by teachers for HIV/AIDS-affected children. Whilst teachers were well aware of the challenges facing such children, numerous factors constrained them in openly acknowledging or responding. In contrast to the Western literature on caring teachers, focusing on the respectful empowerment of vulnerable children through dialogue and responsiveness to their needs, rural Zimbabwean teacher-learner relationships were depicted as top-down and teacher-led, with the teacher instructing or disciplining the child. Support was often limited to providing deprived children with books or pens. Inter-personally, teachers appeared distant from pupils, with little sign of the forms of caring and empathic emotional engagement presupposed in the caring schools literature.

Having explored teachers’ representations of children’s problems and their caring responses, we turn to examine the wider communities in which schools were located. To what extent did wider communities support an ethos of care by teachers?

### Community representations

Policies generally advocate that schools work closely with communities in supporting HIV/AIDS-affected children. How did community members view their responsibilities towards such children? In focus groups, they said many individual community members were assisting individual children of relatives. Some managed to provide support, but others–especially grandmothers of orphans–struggled. They also spoke of some children living alone with no adult support at all.

*They face hunger*, *there is good education in school but they have no one to help them pay their fees*. *They don’t have clothes that make them look like normal children*. *CA05*

*People in the community no longer care about orphans*. *They know that there are orphans living there but I have never seen anyone go to see if they have slept well*. *CA01*

Few community members felt responsibility for assisting children that were not blood relatives, saying there was little they could do given their own poverty and difficulties. Some said that in a situation where they were often not able to feed, clothe or pay school fees for their own children, helping an orphan was simply beyond their means, even with the best will in the world.

Others said too many children needed help. Helping a single child would make little impact on the overwhelming ‘flood’ of need. They also spoke of weak community solidarity in the unstable economic and political context, which fostered social division and suspicion rather than cohesion. People often looked down on AIDS-affected families, whispering that they had brought their problems on themselves and were thus undeserving of help.

*When HIV-affected children are excluded from school*, *we just look at each other and say*: *‘The parents are the ones who are to blame*, *not us*.*’ That is why people fail to help each other*. *They look down upon each other*. *They say*: *‘They brought the problem upon themselves’*. *CA04*

Others argued that HIV/AIDS-affected children were no more needy than others, disagreeing that child carers should be forgiven for arriving late at school or for frequent absences. Many said such actions were nothing more than ‘bad behaviour’, with children deserving any punishment they got. Some expressed strong hostility to the notion of ‘children’s rights’ associated with child-centred foreign NGOs, which strongly contradicted local preferences for hierarchical adult-child relationships associated with obedience, discipline and punishment.

As with teachers, community members who might have wanted to help HIV/AIDS-affected children said well-intended acts of kindness risked unwittingly causing offence, with HIV/AIDS-affected families misinterpreting them as stigmatising or patronising, or suspecting helpful outsiders of trying to bewitch them.

*One day l met a boy who was wearing torn trousers*, *I told him to ask his grandmother to come and get some spare trousers from me for his school uniform*. *When I met the grandmother she coldly refused to greet me and told me never to do that again*. *If you try to help someone they may think you are making fun of their poverty*. *CB01*

*If people hear that I gave a child a jacket they will say I am the one causing misfortunes to the child*, *I will be accused of being a witch*. *I will be accused of causing the poverty they suffer at that child’s home*. *So I cannot try to help in that kind of situation*. *CB01*

NGO support for HIV/AIDS-affected children included help with fees by community-based organisations, BEAM and sometimes churches. This support was often short term and fluctuating, and since the time of our study, BEAM funding–which people repeatedly referred to as a lifeline–has been substantially reduced. Local state clinics provided free AIDS medication to children, and community members spoke of one-off community gardening initiatives providing food for children and sometimes teaching them gardening skills. There were virtually no references to local community groups (e.g. women’s or youth groups) providing help or support.

Community members perceived schools as having little genuine interest in the well-being of HIV/AIDS-affected children. They said teachers’ overwhelming motivation was to extract cash incentives from children to supplement their low government salaries, sending children home if they were unable to pay (despite this being illegal). Some refused to teach pupils who failed to provide these, making them sit at the back of the class and sometimes face the opposite wall.

*If you ask the teacher to allow your child to attend school even though you can’t pay the incentive*, *the teacher will agree to your face*. *But when your child goes to school he will be told to sit with his back to the teacher and to the black board*. *The teacher will teach the children who paid [the cash incentive]*. *The children who did not pay will be told to teach each other*. *CB01*

However community members did make several references to acts of kindness by individual teachers or principals, whilst emphasising that their ability to help was constrained by the determination of many families to deny that they were HIV/AIDS-affected.

Formal efforts to promote opportunities for school-community liaison were seen as unsuccessful. Local School Development Committees, mandated to mediate between parents and schools, were regarded as toothless, with parent representatives only concerned with advancing their own children. Efforts to set up parent-teacher meetings were also not seen as effective, with many less educated parents unfamiliar with the notion of parental involvement in children’s education, or family engagement with schools.

Despite many shortcomings, however, our community focus groups unambiguously represented school attendance and education as the greatest good for children and communities. Participants made lengthy and detailed references to the positive role of schooling in the moral development of youths, providing not only knowledge and training for adult employment, but also preparing them for adult citizenship and fuelling their future hopes and dreams.

*If a child goes to school he will be safe and also if a child goes to school even if he is not good in school he can think rationally or think of ways that can make him earn a living rather than a person who dropped out of school*. *If you go to school you can have a good life and take care of your parents*. *A child who goes to school is well mannered and knows what to with his life when he finishes school*. *Going to school is good so that you can be able to sustain yourself in the future*. *CA02*

Children out of school were depicted varyingly as immoral, both in relation to sex and drugs, more likely to be cruel or bad mannered, and vulnerable to abuse. All agreed that regular school attendance was necessary for HIV/AIDS-affected children to have comparable life opportunities as other children. School attendance was seen to increase their opportunities to access NGO and other forms of assistance, and to distract them from the worries of their daily lives.

## Discussion

To what extent did schools in our study support key dimensions of international school-related policy: psycho-social support, framed by strong community-school partnerships and robust alliances with outside health and welfare agencies, using empowerment-based approaches? What did our findings suggest about the extent to which schools can ‘substitute for families’ in supporting the school inclusion and the emotional and physical well-being of HIV/AIDS-affected children [6]?

What is the wider ethos of care that discourages concerted, widespread and effective action by teachers? What possibilities exist for transforming the constraints that inhibit caring by teachers? Many arise from the political and economic contexts of Zimbabwe, unlikely to be conclusively resolved in the short-term future. What schools can do with current resources, in the difficult settings in which many African people with HIV live, and in the absence of significant outside support?

Our research design and our interpretation of the resulting empirical data was framed by the inter-linked conceptualisations of care as a social relationship, enacted in schools conceptualised as social spaces constituted through the interaction of groups (including teachers, pupils and members of the surrounding communities) who often had very different understandings of children’s needs and interests and of the nature of their responsibilities in responding to these. In our empirical findings section above we have sought to illustrate the ethic of care that had arisen in the wider contexts of schools and their surrounding communities. Relevant dimensions of context included the impacts of poverty and stigma in wider contexts of political uncertainty, the associated lack of trust and mutual support within the communities surrounding schools, and the associated institutional strain within schools and its impact on teacher identities.

In this discussion section we use our conceptualisation of the ‘HIV-competent community’–developed in our earlier work on community responses to HIV and AIDS in southern Africa [46,47]–to systematise our understandings of the obstacles to an effective ethic of care in our case study schools. HIV-competent communities are local contexts where people are most likely to work collaboratively to provide optimal care and support to the HIV/AIDS-affected [40]. The three aspects of HIV-competence most relevant to our interests in this paper are: a group’s confidence in their skills and ability to contribute to a more effective HIV response; a sense of solidarity and common purpose in relation to tackling HIV/AIDS, and effective alliances with supportive NGO and public sector agencies. We examine each in turn.

### Teachers’ confidence in their skills and ability to support HIV-affected learners

As reported above, many teachers lacked confidence in their ability to support HIV/AIDS-affected children. Many said they lacked formal skills (e.g. knowledge about AIDS, how to talk to children about sex, counselling skills). Teachers defined their role predominantly in terms of the transmission of factual knowledge and the exercise of discipline, with the notion of care not fitting easily into this framework. Many were overwhelmed by the extent of children’s needs relative to the lack of resources available.

Several teachers faced overwhelming problems in their own lives. Although none disclosed positive status to us estimates suggest that one in three are likely to have been HIV-positive themselves [9], and struggling to solve HIV-related problems in their own families [14], let alone feeling able to tackle complex issue of counselling and supporting the highly marginalised children in their care. Above, we have cited Noddings’ [24] US-based work which emphasises the need for teachers to ‘emphathise’ with children’s problems as the pathway to caring attitudes and behaviours. Our Zimbabwean findings suggest that effective empathy of this sort might require a degree of distance between the lives and problems of teachers and children, and a degree of social confidence on the part of the teachers arising from their own experience of having solved problems in their own lives. In the absence of such distance and confidence, the potential for the development of such caring might be significantly undermined,

At the institutional level, our study highlighted the lack of teacher awareness of any clear caring policy in schools and the absence of time and workload allocation for this additional work. As embattled school principals struggled to keep their schools open under often almost impossible conditions, HIV/AIDS-affected children were only one of an avalanche of demands they faced.

### Solidarity and common purpose between teachers and learners in responding to children’s problems

We have highlighted the disconnect between children’s emphasis on the emotional challenges facing HIV/AIDS-affected peers [39–41] and teacher emphases on children’s needs for material support and discipline. This would have undermined teacher-pupil solidarity in jointly identifying and responding to co-constructed understandings of children’s needs. A further obstacle was teacher views of teacher-pupil engagements as hierarchical, defining their pastoral roles in terms of rules, discipline and punishment. Such an ethos mirrors hierarchical child-adult relationships typical of many southern African communities [48]. In our study it was said to limit opportunities for pupils to share their worries with their teachers, or for teachers to provide empathetic emotional support of the type emphasised in the western ‘caring schools’ literature. In this respect the situation was a far cry from the international policy emphasis on children’s rights and empowerment in the context of equal and dialogical relationships between teachers and learners. The situation was further undermined by high levels of HIV denial and stigma, which often made it impossible for teachers and pupils to acknowledge the existence of a child’s HIV/AIDS-related problems, even in situations where pupils were clearly suffering deeply.

Overall, the needs and priorities of teachers and children often appeared to diverge in ways that defied mutually empowering negotiation. Given their wider financial insecurities, many teachers were predisposed to view pupils as an income stream best optimised through pressurising children and families to top up their salaries with incentives, and to experience resentment and financial strain when such cash was not forthcoming. This situation was far from the policy assumption that teachers would be able or willing to take on complex *additional* roles (counselling, psycho-support, community liaison) requiring an even greater commitment of time and energy.

Furthermore, teachers’ willingness to take on additional roles was coloured by their deep demoralisation caused by the recent Zimbabwean school closures and irregular salaries, and the generally declining state of the profession. They were also demoralised by the poor physical conditions of their schools, use of alcohol by colleagues at work, the lack of electricity and telephones and so on, hampering their pride in their daily work and their confidence in what they could offer children. Headmasters felt disempowered to exercise motivational HIV-related leadership (or indeed any leadership at all) in such a setting. If caring involves a degree of empathetic engagement with others, one’s ability to empathise is heightened when one stands on a platform of confidence in one’s own social value. Many aspects of teacher’s working lives had dented this confidence.

This is not to say that school attendance was not helpful to pupils. In relation to their material needs, school attendance did indeed seem to provide some HIV/AIDS-affected children with access to particular forms of NGO support, and to individual acts of kindness from individual teachers. In terms of their social needs, our data suggested that schools provided children with what both teachers and the community regarded as valuable discipline, boundaries and guidance on good values and norms for moral behaviour, as well as the social respect accorded to children in school compared to out-of-school youths.

### Effective bridging alliances between schools and community (residents, government, NGOs)

The wider community offered the school little support in terms of a broader community ethos of care for HIV/AIDS-affected children who were not direct family members. There were no effective working structures to promote school-community liaison, and no culture of committed parents engaging with school. Schools operated in a vacuum in the absence of stable and sustained support from external agencies, with reductions in public sector funding and withdrawal of NGOs. In relation to their policy mandate to facilitate the school access and enrolment of HIV/AIDS-affected children, there was some evidence that teachers had in fact referred children to government, NGO and church bodies for help with fees–in so doing, also enacting the ‘liaison’ role outlined for them in policy. However, the availability of support from these external groups fluctuated. Thus, for example, at the time of our interviews teachers said that whilst BEAM had played a key role in providing fees for HIV/AIDS-affected children in the previous two years, the supply of money had dwindled. It appeared that individual schools had policies requiring teachers to keep attendance records, and to follow up (if necessary by home visits) those children who were persistently absent. Only occasional references were made to this practice in the interviews however, with teachers commenting that they were seldom adhered to.

### Creating contexts that support an ethic of care by teachers?

In what ways does international policy resonate with the daily lives and possibilities of teachers in rural Zimbabwe? Can teachers realistically be expected to take on these additional roles? Whilst teachers pointed to ways they might offer such support in principle, in practice few viewed themselves as qualified or willing to take them on. Neither were supports in place to enable them to do so. How might policy prescriptions be expanded to support the development of such supports?

In South Africa, Hoadley has argued that the HIV-related expansion of understandings of the functions of schools and of teaching within dominant policy and NGO discourses over-burdens teachers with the responsibilities that should be taken by other professionals such as social workers, community nurses, counsellors and welfare grant officers [19]. At most, she argues, schools should be viewed as referral networks that facilitate children’s access to appropriate external support, without having to offer care and support themselves. Others have warned that such expansion of roles threaten to divert resources and attention away from the vital academic functions of teaching and learning [49].

However, given the dearth of other institutional resources in rural Zimbabwe, in wider contexts of crippling poverty and stigma, such arguments potentially leave children completely unsupported. Even heavily challenged schools have potentially vital infrastructure. Schools have the greatest daily access to children. More than other adults, teachers are with children all day. Furthermore, our own study did highlight examples where individual teachers overcame obstacles to offer kindness. And our companion studies of children’s experiences of school points to cases where children have shown exceptional resourcefulness in using the meagre resources offered by schools to assist them in day-to-day survival [39–41]. Teachers and schools must be a vital piece of the puzzle.

The challenge then becomes how best to create school contexts that better enable and support an ethic of care amongst teachers. Our study points to three particular aspects of HIV-competence particularly lacking in schools: teacher motivation and capacity for caring; a background context of teacher-pupil solidarity; and adequate bridging networks to support care by teachers. In relation to the first two aspects of HIV-competence, current HIV impact mitigation efforts in southern Africa currently tend to lay heavy emphasis on ‘training teachers to care’. Such training programmes tend to focus on developing individual-level psychological skills in areas such as grief counselling, listening skills, memory boxes and HIV-awareness–paying less attention to the contexts that shape whether teachers are likely or willing to put this training into action. Counter to this trend, and relating to the third aspect of HIV competence mentioned above (creating bridging networks between schools and other support organisations), child welfare networks in South Africa do make some reference to the need for ‘circles of support’ around schools, creating better channels between schools, local health and welfare services, and local community organisations [49]. To date this work is largely confined to the grey NGO literature, however, and is not systematically supported by research.

What do our findings suggest about the types of contextual supports needed for teachers in the Zimbabwean context? What policies and interventions would best facilitate social environments in which it is possible for them to do so? As outlined above, recognition is core to professional identities, and a key factor here would involve formal public recognition both of the value of teachers in general, as well as recognition of the additional burdens their new caring roles entail. In particular, rather than simply targeting teachers, there is a need to turn the spotlight onto the institutional settings that currently provide so little support for their caring roles.

One goal for those seeking to increase teachers’ motivation to take on expanded roles would involve pressurising Education Ministries to fight for better salaries and improved status of teachers in general. Another useful and more easily achievable shorter term goal might be for NGOs and policy-makers to work with Education Ministries to advocate for greater formal recognition of the time and labour intensive nature of caring by teachers. This would be most likely to happen through the systematic incentivising of ministry officials, school principals and teachers to engage in care and protection activities. The existence of formal caring schools policies is not enough–our data suggested that these were often not implemented. Such a strategy might involve working directly with ministry officials and head teachers to think of ways of recognising and elevating caring to a core function of teachers, building dedicated space for caring activities into teacher job descriptions and timetables, making involvement in caring a core precondition for promotions and salary increases and so on.

Caring work might also be supported by the appointment of dedicated ‘socio-emotional support staff’ to support teachers in their caring roles. These might consist of especially trained social workers, each of whom might be delegated responsibility for the on-going support of teachers in a portfolio of schools in a particular region. They might also take responsibility for supporting head teachers in building appropriate ‘circles’ of back-up referral and support around the school.

## Conclusion

We end this paper with some general reflections. Our discussion of our findings in the light of our HIV competent community framework suggests that if the school setting were to provide teachers with greater support, time and recognition for caring work, as well as wider circles of referral networks to health, welfare and local community groupings, teachers might feel more supported themselves and more likely to feel equipped to offer care to children, as well as more motivated to make the effort.

We base this argument on Paul Farmer’s claim that a key driver of HIV stigma and denial within communities and institutions is the lack of resources to respond effectively [50]. People often shy away from or react negatively to those facing problems they feel powerless to help with [51]. People are most likely to reject those who seem the most defenceless and unsupported when there is nothing they can do for them. If teachers had more recognition and resources to respond effectively to HIV/AIDS-affected children, even in little ways, they might be more likely to feel confident to reach out to them. In turn, if children, their families and communities believed that teachers could help them, even in very small ways, they might be more willing to disclose their need for help.

Another major challenge facing those who seek to advance a caring role for teachers in southern Africa is that of developing more culturally appropriate notions of care, that resonate with the more hierarchical and authoritarian notion of adult-child relations reflected by teachers and communities in our study. Local notions of care were often out of kilter with the western emphasis on children’s rights, children’s ‘empowerment’ and children’s entitlement to a sense of social equality with adults outlined in our discussion of the western caring schools literature above [48]. In our context, it was the case that teachers were more familiar with the material and practical dimensions of caring than the provision of emotional support. In such a context, where any increase in material resources is unlikely in the immediate term, it might be worthwhile for ‘caring teachers’ programmes to focus on helping teachers to identify and provide locally appropriate and feasible forms of practical support to HIV/AIDS-affected children as small first steps towards the gradual development of a wider notion of their caring roles.

The inappropriateness of idealised ‘one size fits all’ western notions of caring in the context of the extremely challenging reality of everyday life in Zimbabwe was a constant theme during the production of this paper. It repeatedly emerged in on-going discussions of the empirical material between the paper’s first two European authors (CC and LA), and the Zimbabwean third author and project fieldworker (AM). AM reacted with some irritation to CC and LA’s repeated emphasis on children’s emotional needs. She argued that “in Zimbabwe all our energy is taken up with basic survival, we simply don’t have time to agonise about each others’ emotional needs in the way that you do in Europe”.

In the absence of urgent attention to the obstacles facing teachers in implementing policy prescriptions that they substitute for sick or absent families, there is a danger that the current policy emphasis may be failing HIV/AIDS-affected children through creating a false impression that teachers, schools and local communities are willing and able to shoulder the burden of their care and protection–in settings where unsupportive institutional and social contexts make this so unlikely.

This conclusion is framed by the authors’ wider concerns about the way in which functions traditionally performed by NGOs and public sector are being shifted onto local institutions (such as schools) in the wider context of cuts in development aid and in public sector spending. Heavily marginalised communities and under-resourced and over-burdened schools cannot be expected to single-handedly shoulder problems rooted in inter-twined wider economic, social and cultural dynamics over which they have little control. International policy must be careful that the policy rhetoric of teacher involvement does not serve as a smokescreen to hide the current lack of support for children. Without significantly more support and recognition, the ability of teachers to support HIV/AIDS-affected children remains minimal.
